# Intracranial Myeloid Sarcoma Metastasis Mimicking Acute Subdural Hematoma

**DOI:** 10.1155/2017/3056285

**Published:** 2017-10-22

**Authors:** Amandip S. Gill, Rabina Gill, Paul Kaloostian, Dina Elias, John S. Roufail, Aurora S. Cruz, Panayiotis E. Pelargos, Frank P. K. Hsu, Ronald C. Kim, Robert E. Ayer, Samer Ghostine

**Affiliations:** ^1^Heera Neurosurgical Associates, 17075 Devonshire St., Suite 101, Northridge, CA, USA; ^2^University of California, Riverside, Riverside, CA, USA; ^3^University of California, Irvine, Irvine, CA, USA; ^4^Riverside Community Hospital, Riverside, CA, USA

## Abstract

Myeloid sarcoma, a rare consequence of myeloproliferative disorders, is rarely seen in the central nervous system, most commonly in the pediatric population. Although there are a handful of case reports detailing initial presentation of CNS myeloid sarcoma in the adult population, we have been unable to find any reports of CNS myeloid sarcoma presenting as a large mass lesion in a herniating patient. Here, we present the case of a patient transferred to our facility for a very large subdural hematoma. Based on imaging characteristics, it was felt to be a spontaneous hematoma secondary to coagulopathy. No coagulopathy was found. Interestingly, he did have a history of acute myeloid leukemia (AML) diagnosed 2 months previously, and intraoperatively he was found to have a confluent white mass invading both the subdural and subarachnoid spaces. There was minimal associated hemorrhage and final pathology showed myeloid sarcoma. This is the first report we are aware of in which CNS myeloid sarcoma presented as a subdural metastasis and also the first report in which we are aware of this etiology causing a herniation syndrome secondary to mass effect.

## 1. Introduction

Myeloid sarcoma is a rare pathology that is seen as focal tumor in 2% of leukemia patients [1], comprising mainly extramedullary accumulation of immature myeloid cells/myoblasts. These tumors often coincide with diagnoses of acute myeloid leukemia or chronic myeloproliferative diseases [2–6]. However, as such, they typically present in lymphoid organs such as bone, skin, soft tissue, and mucosal linings. These tumors involve the CNS rarely, almost exclusively in the pediatric population. The few case reports in adults describe dural-based or focal parenchymal lesions in patients with nonspecific neurological complaints, responding inconsistently to various treatments [5, 7–9]. Universally, patients tend to have a poor prognosis. Here, we present a case report of a patient presenting with what was thought to be a spontaneous subdural hemorrhage but upon attempted evacuation was found to be a subdural myeloid sarcoma metastasis.

## 2. Case Presentation

A 54-year-old African American male initially presented to an outside hospital complaining of headache, which had been present for two months but in the last week was worsening per patient's family. Two months previously, he had been diagnosed with chronic myeloid leukemia (CML) initially diagnosed to be in the chronic stable phase and had been maintained on hydroxyurea. The patient was admitted to the hospital and order for head CT placed. While on the floor, the patient complained of sudden worsening of headache and eventually became nonresponsive, requiring emergent intubation. Head CT was obtained showing large subdural hematoma (SDH) for which the patient was given 100 grams of mannitol and he was transferred to our facility immediately.

**Figure 1:**
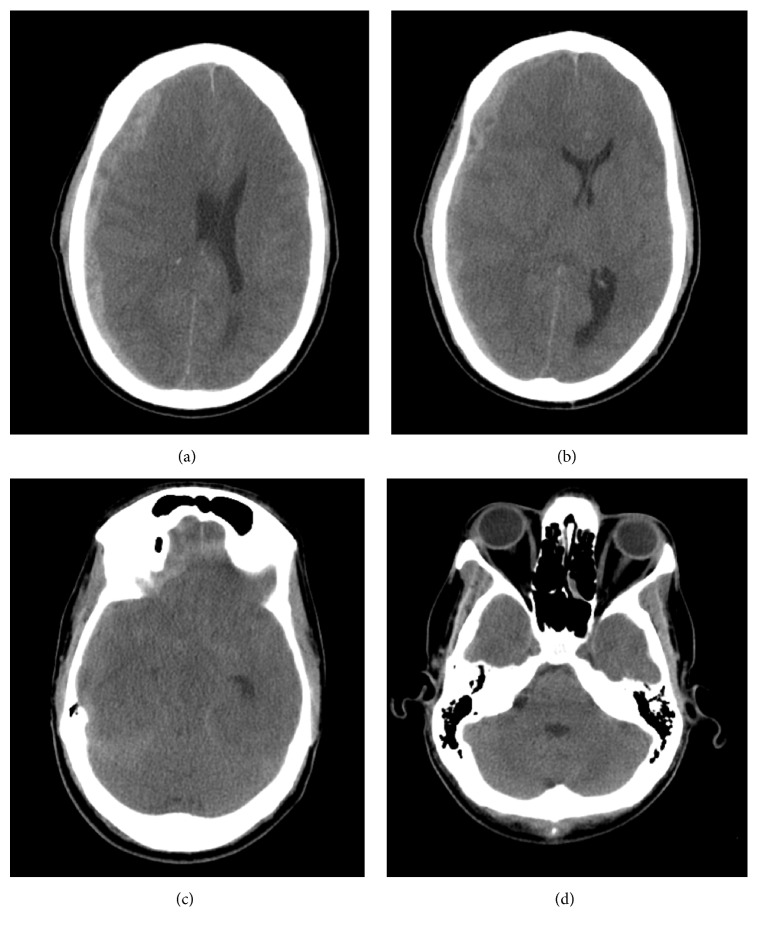
Initial CT scan showing panhemispheric, mixed density subdural mass with significant mass effect ((a) and (b)). There is significant effacement of the right temporal horn and basilar cisterns consistent with herniation (c), though the 4th ventricle is still patent (d).

Upon intake, the patient was noted to have a fixed and dilated right pupil, reactive left pupil, and trace movement in his right upper extremity. Head CT (Figure 1) showed 1.5 cm right panhemispheric hyperdensity in the subdural space with significant midline shift, falcine herniation, uncal herniation, and loss of basilar cisterns. Given the mixed density on imaging and rapid progression symptomatically, outside hospital physician had assumed a spontaneous subdural hematoma secondary to coagulopathy. Interestingly, the patient's platelet count, PT, PTT, and bleeding time were all well within normal limits. The patient was taken emergently for hemicraniectomy and hematoma evacuation.

**Figure 2:**
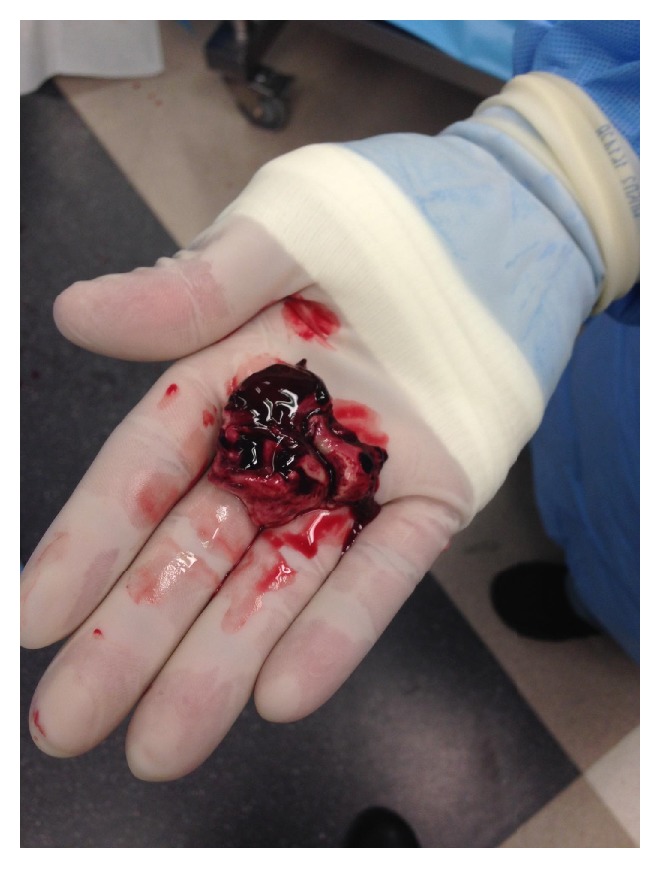
Confluent, white mass seen on initial resection, the pathology of which returned as myeloid sarcoma.

**Figure 3:**
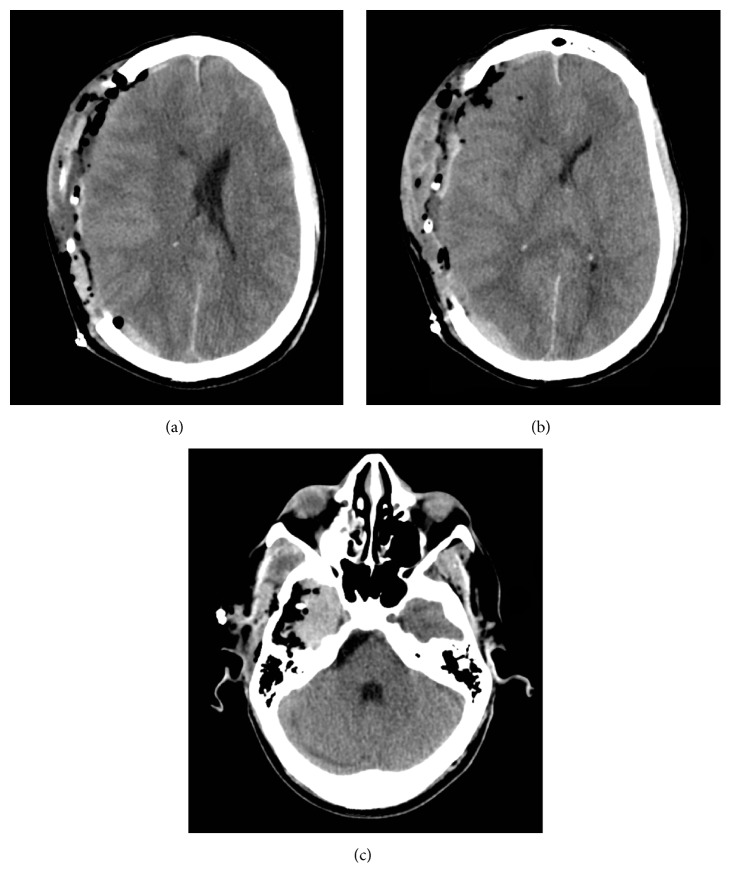
Immediate postoperative head CT showing complete resection of subdural hyperdensity (a) with loss of midline shift seen previously (b) and small subtemporal hematoma (c).

To the great surprise of the operating surgeon, upon dural opening, large clumps of confluent white material expressed themselves under pressure (Figure 2). Very little hematoma was present; copious irrigation throughout both the subdural and subarachnoid spaces revealed more confluent material again with very little blood. Persistent irrigation of the subarachnoid/subdural spaces continued to yield additional confluent white material. After evacuation, the patient had intracranial pressure monitor placed and had a postoperative head CT (Figure 3).

**Figure 4:**
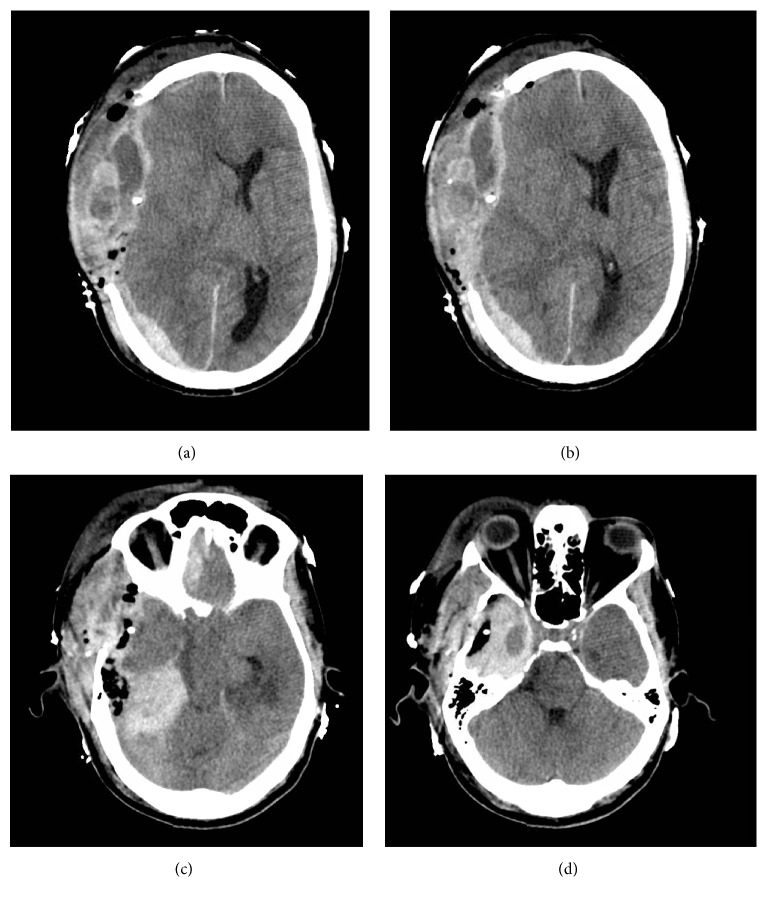
Repeat CT 6 hours after first surgery showing reappearance of mixed density mass along the right convexity with again persistent midline shift ((a) and (b)). There is reherniation with loss of right temporal horn and basilar cisterns once again (c). Of note, the imaging characteristics of the small subtemporal hematoma are unchanged (d), speaking to the differing contents of the panhemispheric subdural density (versus hematoma) seen in panels (a) and (b).

**Figure 5:**
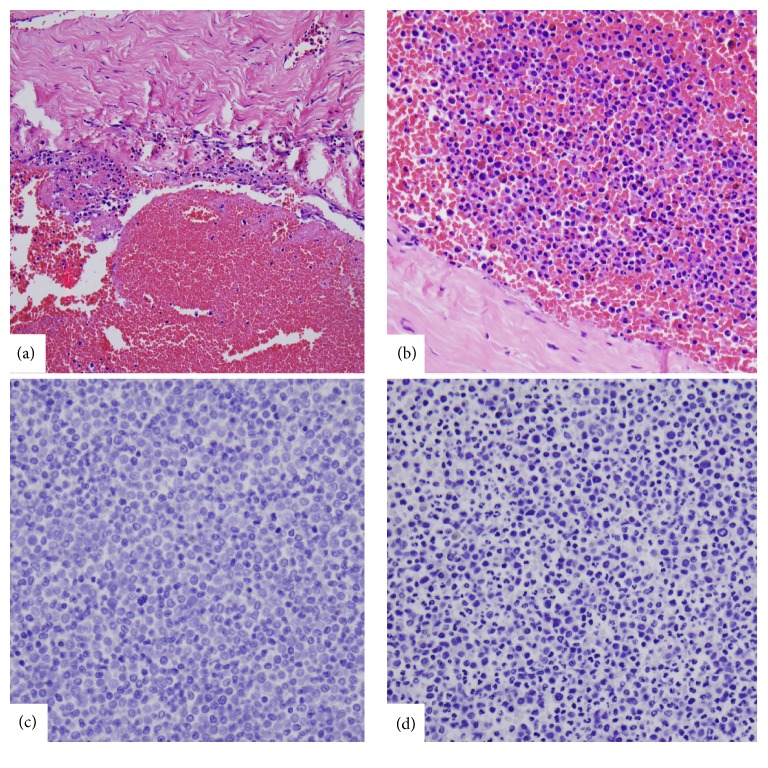
Photomicrographs: low-power (a) and high-power (b) H&E stains showing lymphocytic invasion throughout the subdural space. Panels (c) and (d) show confluent immature myeloblasts consistent with myeloid sarcoma.

The patient was initially kept sedated in the ICU and had good ICP control for roughly 6 hours. Thereafter, he began having progressively worsening ICP spikes, at which point repeat head CT was done (Figure 4), once again showing large accumulation of subdural mass. As his exam never improved from presentation, his family was counseled as to likely futility of reoperation, though eventually they requested evacuation. Second decompression again revealed confluent mass in the subdural and subarachnoid spaces with very little hematoma. After second evacuation, the patient continued to have malignant intracranial hypertension with no improvement in clinical exam. His family chose to withdraw care 3 days later. Pathology revealed myeloid cells with immunohistochemistry staining negative for CD34 and CD117, consistent with mature myeloid sarcoma (Figure 5).

## 3. Discussion

Myeloid sarcoma is a very rare pathology most often seen in patients with preexisting myeloid disorders [1]. The disease is most often seen in the pediatric population, though a few case reports in the adult literature describe focal lesions in patients with vague neurological complaints [5, 7–10]. In this unfortunate case, the patient presented with headache in the setting of known CML. As the patient's neurological status deteriorated, head CT revealed panhemispheric right-sided hyperdensity suspicious for subdural hematoma. In retrospect, the mixed density seen on imaging was secondary to tumor rather than hyperacute SDH. Two surgeries revealed gross tumor invading the subdural and subarachnoid spaces.

Myeloproliferative masses in general tend to mimic lymphoid tissue microscopically [1, 8, 10]. The operating authors feel that most likely the patient had dural-based metastases of his CML, which then began producing the white, confluent, lymph-like material seen upon surgery. This led to a rise in intracranial pressure that eventually led to the herniation syndrome described. Interestingly, there was a rapid accumulation of subdural material after the initial surgery. The authors hypothesize that this tumor metastasis was likely mimicking myeloid and lymphoid tissue, expressing exudate which eventually stopped as the intracranial pressure rose to malignant levels. After evacuation and copious irrigation of the subarachnoid and subdural spaces, the remainder of the metastatic tumor not immediately underneath the craniectomy site continued to express this tumor-laden exudate, which now rapidly accumulated given the rapid and extensive decrease in intracranial pressure. 6 hours later, this led to a significant reaccumulation of the mass, accumulation accentuated by the lack of resistance over the right hemisphere given the hemicraniectomy. As seen grossly and as in Figure 4, the contents of the subdural material were very different from hematoma which was visible on the same cut on the CT scan as a small, residual subtemporal hematoma after the first surgery.

This presentation is unique not only in that it is the first report of CNS myeloid sarcoma presenting in the subdural space, actually mimicking a large subdural hematoma, but also in that the lesion showed significant mass effect, which eventually resulted in herniation of the patient. As chemotherapeutic agents result in greater life expectancies across an array of cancers, subdural metastases are more frequently being encountered by neurosurgeons and oncologists alike. This case report also highlights the severity of the prognosis of the condition, despite early and aggressive management.
